# Overexpression of Pleomorphic Adenoma Gene-Like 2 Is a Novel Poor Prognostic Marker of Prostate Cancer

**DOI:** 10.1371/journal.pone.0158667

**Published:** 2016-08-18

**Authors:** Jia Guo, Min Wang, Zhishun Wang, Xiuheng Liu

**Affiliations:** Department of Urology, Renmin Hospital of Wuhan University, Wuhan University, Wuhan, Hubei, PR China; University of Colorado Denver, UNITED STATES

## Abstract

Pleomorphic adenoma gene like-2 (PLAGL2) is a member of the PLAG gene family. Previous studies have revealed that overexpression of PLAGL2 is associated with many human cancers. However, it has been reported that PLAGL2 also plays as a tumor suppressor. The precise role of PLAGL2 in prostate cancer (PCa) is still unknown. The aim of this study was to investigate the expression and prognostic value of PLAGL2 in PCa. Data from microarray datasets demonstrated that the DNA copy number and mRNA level of PLAGL2 were significantly increased in PCa compared with normal prostate. qRT-PCR and western blot analysis from paired PCa samples and prostate cell lines confirmed upregulated mRNA and protein expression levels in PCa. Immunohistochemistry analysis showed that staining of PLAGL2 in PCa tissues was significantly higher than that in benign prostatic hyperplasia (BPH) tissues. In addition, the high expression of PLAGL2 was only involved in preoperative PSA, but was not related to age, Gleason score, seminal vesicle invasion, surgical margin status, clinical stage and positive lymph node metastasis. Moreover, our results showed that PLAGL2 was an independent prognostic factor for biochemical recurrence (BCR)-free survival and overall survival (OS) of PCa patients, and overexpressed PLAGL2 was related to early development of BCR and poor OS. In conclusion, our findings suggest that PLAGL2 is overexpressed in PCa. The increased expression of PLAGL2 correlates to PCa progression following radical prostatectomy and may serve as a novel poor prognostic marker for PCa.

## Introduction

In the developed countries, prostate cancer (PCa) has been proved to be the second most common cancer, which has become one of the leading causes of cancer death in men [1]. More PCa patients have been diagnosed at younger ages in recent years [2]. There are already many treatment options for localized PCa, including radical prostatectomy, hormonal androgen deprivation therapy, radiotherapy, brachytherapy, and active surveillance [3]. Although the prognosis is good for patients with localized or regional PCa, there are cases of aggressive PCa which could metastasize primarily to the bone [4, 5]. In the United States, the 5-year survival rate of the patients who develop metastatic disease is only 29% [6]. There are no reliable biomarkers for prediction or effective therapies of metastatic PCa.

To date, prostate-specific antigen (PSA) is still the most commonly used tool for screening and early detection of PCa. Although diagnostic tools, including pretreatment PSA, clinical stage and biopsy Gleason score, are all significant for PCa prognosis, there are some limitations of these conventional prognostic factors. Single-cell analyses have revealed that PCa cells are highly heterogeneous [7]. 20–30% of the patients may undergo recurrence five years following initial treatment [8]. The variety in biological behavior of PCa demands identification of biomarkers that may distinguish a slow growing cancer from a more aggressive cancer with the potential of metastasis [9]. For PCa patients, misdiagnosis and overtreatment are major concerns in clinical treatment [10]. To this respect, more efforts are needed to seek new biomarkers of initiation, progression, metastasis and prognosis in PCa.

Pleomorphic adenoma gene like-2 (PLAGL2), a member of the PLAG gene family, is a transcription factor which includes a C-terminal trans-activation domain preceded by 7C2H2 zinc fingers with DNA binding function [11]. PLAGL2 shows high homology and similar DNA-binding affinity with PLAG1 which has importance and versatility of oncogene in tumorigenesis [12]. The expression of PLAGL2 is found ubiquitously in almost all adult human tissues [13]. Previous study showed that PLAGL2 was an essential genetic event in leukaemogenesis and participated in AML development [11, 14]. Moreover, accumulating reports indicate that PLAGL2 is implicated in the progression of various cancers, including malignant glioma, lung adenocarcinoma, breast, gastric and colorectal cancer [11, 15–20]. It is interesting that PLAGL2 possesses both oncogenic and tumor suppressive activity. PLAGL2 can induce cell cycle termination and apoptosis [21]. Thus, despite the previous efforts, the precise role of PLAGL2 in cells is still largely unknown.

To our knowledge, little is known about the exact expression of PLAGL2 in human PCa. Here, we investigated the distribution of PLAGL2 in clinical human prostate tissues as well as PCa cell lines. The aim was to enlighten the underlying involvement of PLAGL2 expression in PCa. Overall, our study showed that the expression of PLAGL2 was significantly increased in PCa. Furthermore, the overexpression of PLAGL2 was correlated with PCa tumor progression and might serve as a poor prognosis maker following radical prostatectomy.

## Materials and Methods

### Patients and Tissues

The protocol was approved by the Ethics Committee of Renmin Hospital of Wuhan University (Jiefang Road 238, Wuhan 430060, Hubei PR, China), and the written informed consent was obtained from all patients. For qRT-PCR and western blotting analysis, 25 frozen paired PCa and adjacent normal prostate tissue samples were collected from the patients between September 2011 and June 2014. 79 archived formalin-fixed and paraffin-embedded tissue samples were harvested from PCa patients and 80 from benign prostatic hyperplasia (BPH) between January 1999 and December 2009, which were used to examine the immunohistochemical differences.

PCa patients received radical prostatectomy and the BPH patients underwent transurethral resection of prostate (TURP) in Department of Urology, Renmin Hospital of Wuhan University, Wuhan, China. None of the PCa patients received chemotherapy, immunotherapy, or radiotherapy before surgery. PCa patients were followed after surgery by their surgeons from the date of surgery to the date of death or the last follow-up. The follow-up times were recorded for the 5-year survival analysis. PCa biochemical recurrence (BCR): progression with a serum PSA concentration ≥0.2 ng/ml increasing over a 3-month period. Clinical and pathological information of the 79 PCa patients are provided in Tables 1 and 2.

**Table 1:** Expression of PLAGL2 in prostate specimens.

Groups	n	High expression	%	P
BPH	80	7	8.75	<0.0001
PCa	79	58	73.42	

**Table 2:** PLAGL2 expression and clinicopathological variables in 79 PCa patients.

		PLAGL2 expression			
Variables	Group	N	Low	High	p-value
Age	<60	33	9	24	0.906
	≥60	46	12	34	
Gleason	<7	17	7	10	0.124
	≥7	62	14	48	
T stage	1	50	17	33	0.050
	2 or 3	29	4	25	
PSM	negative	29	7	22	0.708
	positive	50	14	36	
PSA	<10	34	13	21	0.042
	≥10	45	8	37	
Extraextension	negative	36	11	25	0.465
	positive	43	10	33	
SV invasion	negative	69	20	49	0.207
	positive	10	1	9	

### Cell Lines

Human prostate cell lines RWPE-1, LNCaP, DU145 and PC-3 were obtained from American Type Culture Collection (ATCC). The benign epithelial prostate cell line RWPE-1 was maintained in keratinocyte serum free media (Gibco). LNCaP, DU145 and PC-3 grown in RPMI 1640 (Life Technologies, CA) with 10% FBS (Invitrogen). All the cells were placed in a 5%CO_2_ cell culture incubator.

### RNA extraction and qRT-PCR analyses

Total RNA was extracted from prostate tissues and prostate cell lines using TRIzol reagent (Invitrogen, Grand Island, NY, USA) and cDNA was prepared using PrimeScript RT Reverse Transcriptase reagent Kit (Takara, Otsu, Japan). Quantitative RT-PCR (qRT-PCR) was performed on the Applied Biosystems 7300 real-time PCR system using SYBR Premix Ex TaqTM (Takara). Expression data were normalized to beta-actin. The PLAGL2 fold changes between PCa and normal tissue pairs were analyzed by calculating the 2^-ΔΔCt^ values. The same method was used for PCa cell lines and RWPE-1. Each sample was run in triplicates.

### Western blot analysis

Total proteins from prostate tissues and prostate cell lines were extracted. The protein concentration was determined using BCA Protein Assay Kit (Merck). 40μg lysates underwent electrophoresis in the 10% polyacrylamide gel, which were then transferred onto nitrocellulose membranes. The rabbit polyclonal anti-PLAGL2 antibody (Abcam, Cambridge, USA) and the HRP-conjugated anti-rabbit antibody (Abcam, Cambridge, USA) were used to probe Western blots. Mouse monoclonal anti-β-actin antibody (Cell signal technology, Boston, USA) and HRP conjugated goat anti-mouse IgG antibody (Cell signal technology, Boston, USA) were used to detect β-actin. Finally, immunoblotting signals were analyzed using Image Station 4000R PRO scanner (CareStream Health, U.S.A.).

### Immunohistochemistry

The four-micrometer sections from formalin-fixed and paraffin-embedded tissue blocks were dewaxed in xylene and rehydrated with ethanol. The tissue sections were incubated in 0.01M citrate buffer (pH 6.0) for antigen retrieval. After blocked in goat serum, the sections were incubated with the anti-PLAGL2 polyclonal antibody (Abcam, Cambridge, USA) overnight at 4°C. Immunodetection was achieved using Vectastain ABC Elite Kit (Vector Laboratories, Burlingame,CA), and sections were counterstained with hematoxylin.

### Immunohistochemical Scoring

By microscopic assessment, the PLAGL2 expression with immunostaining is positive in nuclei. Two pathologists who were blinded to clinicopathological outcome evaluated immunostaining reactions using a semi-quantitative scoring system. The score about the percentage of positive cells (0, ≤5%; 1, 5–50%; 2, >50%) and the score about the staining intensity (0, no staining or weak straining; 1, moderate straining; and 2, strong straining) were combined. All the cases were divided into low expression (0 to 2) and high expression (3 to 4) [22] according to the total score. If the total score was different between the two pathologists, an agreement was reached by rescoring the slides together with a third pathologist on a multi-viewer microscope.

### Statistical analysis

Statistical analyses were performed by SPSS 18.0 software. Student’s t-test was performed for continuous variables and the chi-square test was used to analyze the differences of categorical variables. Univariate survival analysis was carried out by the Kaplan-Meier method and evaluated using the log-rank test. Multivariate Cox regression analysis was used to detect the independent predictors of survival. P < 0.05 indicated significant difference.

## Results

### The expression of PLAGL2 in clinical specimens from publically available microarray datasets

**Fig 1:**
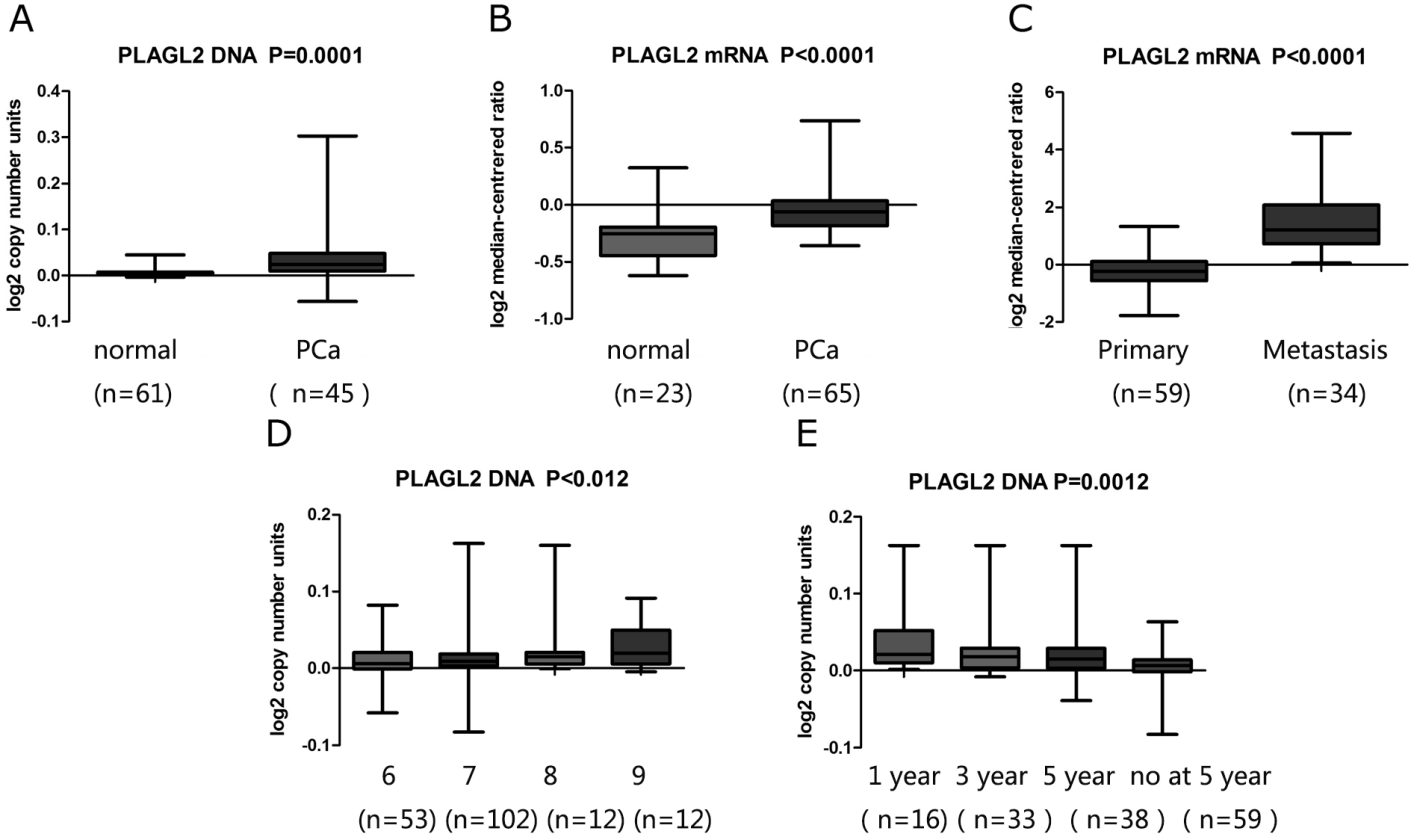
Analysis on PLAGL2 expression in PCa and normal prostate tissues with microarray datasets of PCa and log2 copy number unites of DNA or log2 median intensity mRNA. (A, B) The PLAGL2 DNA copy number and mRNA level was significantly higher in PCa tissues than non-tumor prostate tissues. (C) The PLAGL2 mRNA level was significantly higher in metastatic PCa compared to primary PCa patients. (D) The PLAGL2 mRNA level was significantly increased in higher Gleason score compared with lower Gleason score. (E) The PLAGL2 DNA copy number was significantly higher in earlier recurrence of PCa patients.

Whether there were any deregulated differences of PLAGL2 DNA copy number or mRNA levels in primary and metastatic PCa compared to normal prostate tissues was tested using the data in human PCa from some publically available microarray datasets (https://www.oncomine.org/) [23–25]. The DNA copy number of PLAGL2 was greatly increased in PCa compared to normal prostate (Fig 1). Similarly, the results showed that the expression of PLAGL2 mRNA was significantly higher in PCa than in normal prostate (Fig 1). Additionally, the expression of PLAGL2 mRNA was elevated significantly in metastatic PCa compared to primary PCa (Fig 1). It was also found that PLAGL2 was significantly overexpressed in patients of higher Gleason score than those of lower Gleason score (Fig 1). Furthermore, a strong association between the PLAGL2 DNA copy number and time of PCa recurrence was identified (Fig 1).

### The expression of PLAGL2 in paired PCa and prostate tissues revealed by qRT-PCR and western blot

**Fig 2:**
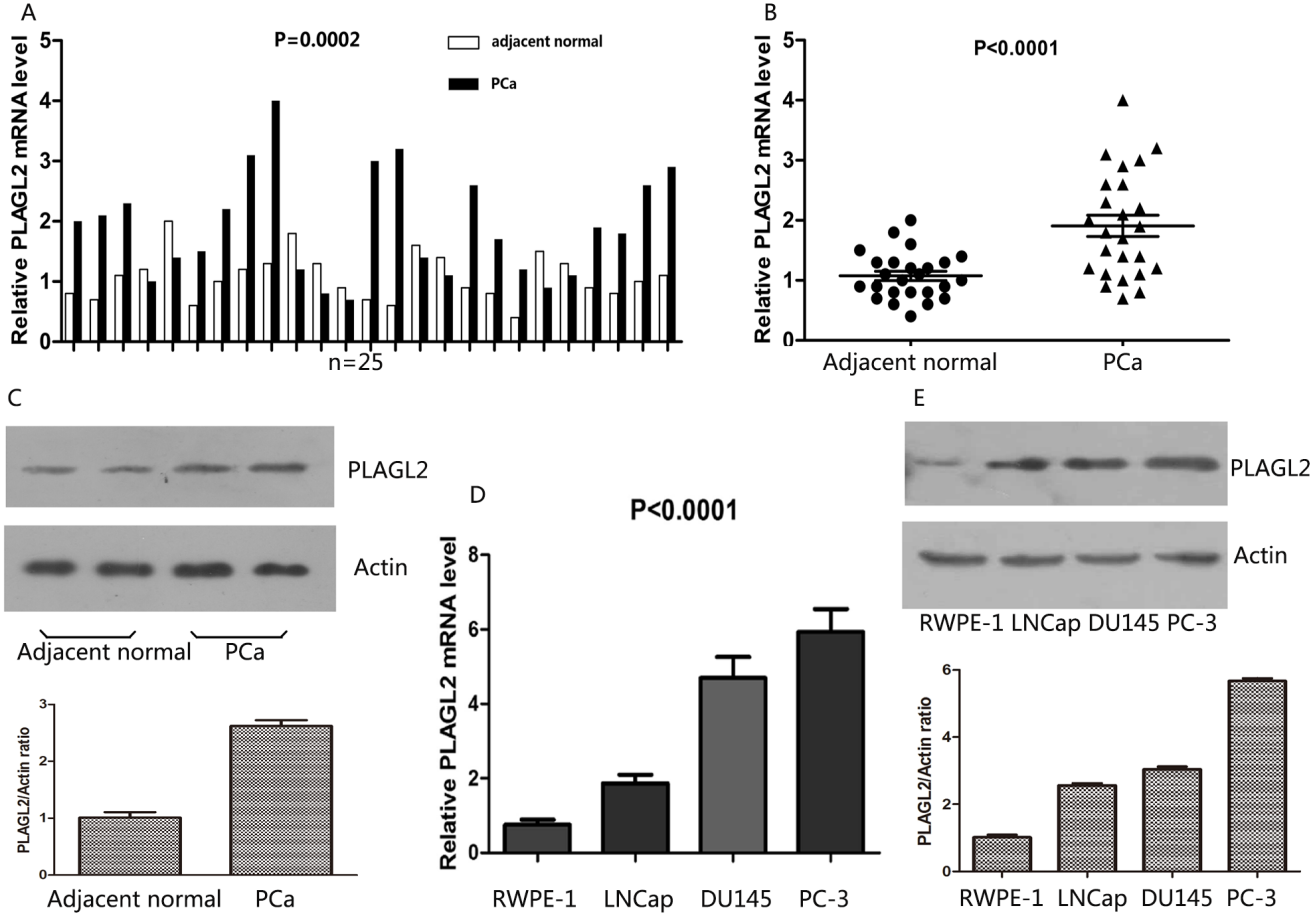
Expression of PLAGL2 in paired PCa with adjacent normal prostate tissues and cell lines revealed by qRT-PCR and western blot. (A) Levels of PLAGL2 mRNA in 25 PCa tissues were significantly higher than those in adjacent noncancerous tissues measured by qRT-PCR (P<0.0001). (B) The PLAGL2 protein level in PCa tissues was upregulated in comparison with adjacent noncancerous tissues measured by Western blot. (C) Western blot indicated the PLAGL2 protein showed higher expression in PCa tissues than in their adjacent normal counterparts. (D) The expression of PLAGL2 mRNA was higher in PCa cell lines (LNCaP, DU145 and PC3) than in RWPE-1 (P<0.0001). (E) Western blot indicated up-regulation of PLAGL2 protein in PCa cell lines (LNCaP, DU145 and PC3) in comparison with RWPE-1.

To validate the distinctly upregulated pattern of PLAGL2 in PCa, mRNA expression of PLAGL2 was assessed in 25 pairs of PCa tissues and adjacent normal prostate tissues by quantitative RT-PCR (qRT-PCR) assay. PLAGL2 was upregulated in 72.0% (18/25) tumor tissues compared with the paired normal prostate tissues (Fig 2). On average, the PLAGL2 expression was significantly higher in tumor tissues than in adjacent normal tissues (P < 0.0001) (Fig 2). As shown in Fig 2, the PLAGL2 protein also showed higher expression in PCa tissues compared to their adjacent normal counterparts, revealed by western blotting analysis.

### The expression of PLAGL2 in prostate cell lines revealed by qRT-PCR and western blot

To further confirm the upregulated expression of PLAGL2 in PCa, we performed qRT-PCR and western blot in three PCa cell lines including LNCaP, DU145, PC-3 and normal prostate epithelial cell lines (RWPE-1). As shown in Fig 2, the PLAGL2 expression was significantly higher in PCa cell lines than in RWPE-1, revealed by qRT-PCR. Similarly, Western-blotting analysis using the PLAGL2-specific antibody showed the higher expression in the PCa cell lines, especially in DU145 and PC-3, compared to the RWPE-1(Fig 2).

### Overexpressed PLAGL2 protein in PCa tissues compared to BPH revealed by IHC

**Fig 3:**
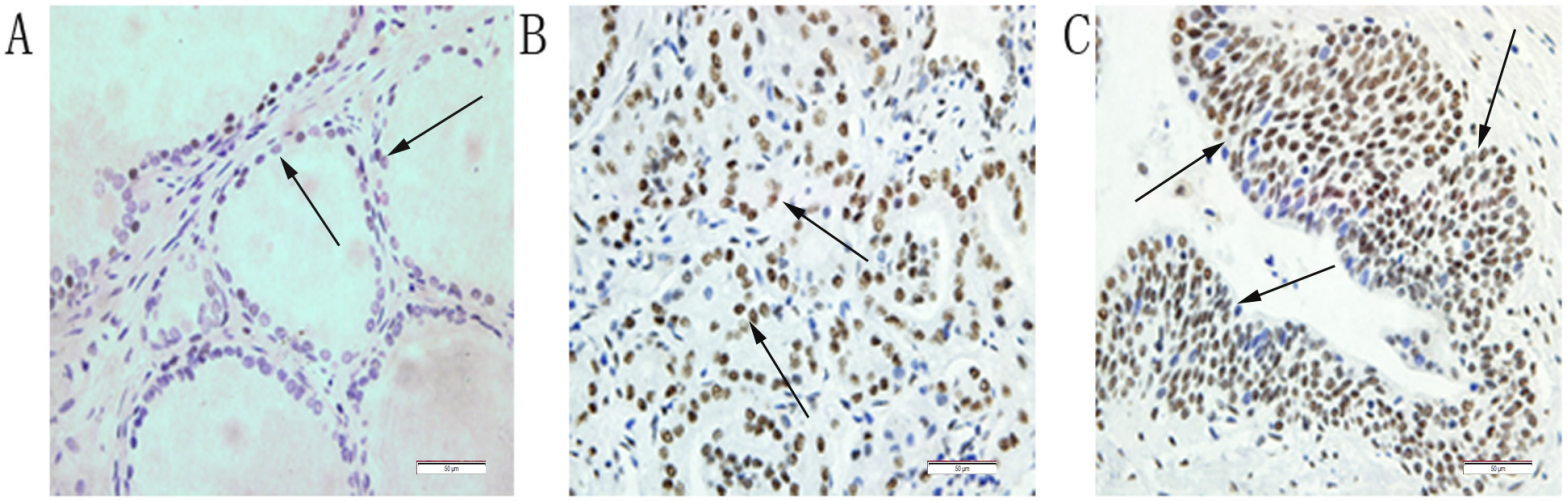
Immunohistochemical staining for PLAGL2 in PCa tissue and BPH tissue. The PLAGL2 expression was predominantly localized in nuclei (positive staining indicated by arrows). (A) BPH tissue (low staining). (B) PCa tissue (low Gleason Score). (C) PCa tissue (high Gleason Score). ×400 magnification.

Immunohistochemistry was performed in all 79 paraffin-embedded and archival PCa tumor samples and 80 BPH samples. The PLAGL2 expression was predominantly localized in nuclei with some also localized in the cytoplasm, and the expression level was categorized as low or high expression (Fig 3). High expression was detected in 8.75% of BPH samples while 73.42% in PCa (Table 1). The protein expression level of PLAGL2 was significantly higher in PCa tissues than in BPH tissues (P<0.0001).

### Relationship between PLAGL2 expression and clinicopathological characteristics of PCa patients

The high PLAGL2 expression was only significantly correlated with the higher preoperative PSA. However, no significant association was observed between PLAGL2 expression and age, Gleason score, seminal vesicle invasion, surgical margin status, clinical stage and positive lymph node metastasis (Table 2).

### Overexpression of PLAGL2 was associated with poor prognosis of PCa patients

**Fig 4:**
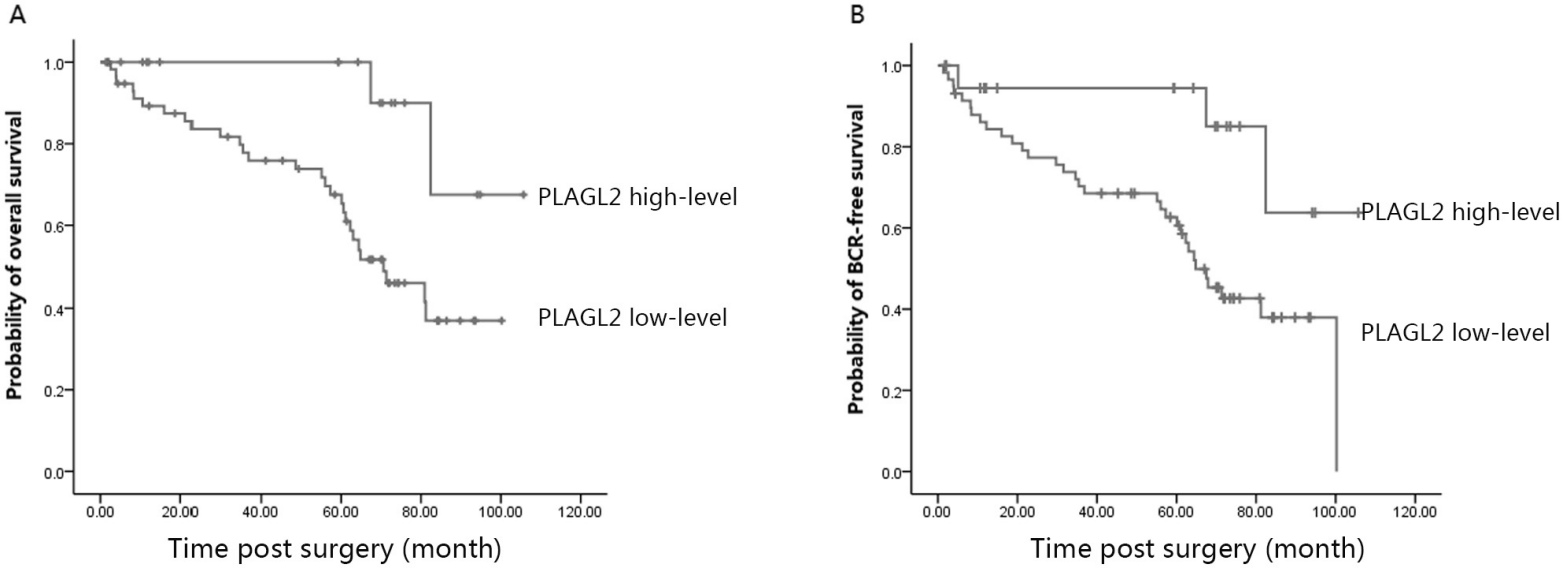
Association between PLAGL2 expression and BCR-free survival and OS of PCa patients assessed by Kaplan–Meier survival curves. (A) The patients with high expression had significantly shorter median 5-year BCR-free survival than those with low PLAGL2 expression. (B) The patients with high expression had significantly shorter median 5-year OS than those with low PLAGL2 expression.

To investigate the effect of PLAGL2 overexpression on the BCR-free survival time and the overall survival (OS) of PCa patients, univariate survival analysis was performed. As shown in Tables 3 and 4, clinical stage, seminal vesicle invasion, surgical margin status, and the PLAGL2 expression were significantly associated with both early development of PCa and poor OS. Next, results of multivariate Cox regression analysis suggested clinical stage, seminal vesicle invasion, surgical margin status, and the PLAGL2 expression were independent prognostic factors for BCR-free survival and OS of PCa patients. Then, the effect of the PLAGL2 expression on the BCR-free survival and OS of PCa patients was further determined with Kaplan-Meier survival curves. Survival length was determined from the day of primary tumor surgery to the day of death or the last follow-up. As shown in Fig 4, the patients with high expression had significantly shorter median 5-year BCR-free survival and OS compared to those with low PLAGL2 expression.

**Table 3:** Prognostic value of PLAGL2 expression for the BCR-free survival revealed by univariate and multivariate analyses.

	Univariate analysis			Multivariate analysis		
Variables	Exp(B)	95%CI	p-value	Exp(B)	95%CI	p-value
Age	1.846	0.916–3.721	0.087			
Gleason	1.914	0.789–4.642	0.151			
T stage	2.435	1.239–4.786	0.010	2.271	1.116–4.619	0.024
PSM	2.018	0.940–4.331	0.072			
PSA	0.965	0.484–1.922	0.919			
Extraextension	3.729	1.798–7.731	0.000	3.188	1.510–6.729	0.002
SV invasion	5.993	2.438–14.731	0.000	4.758	1.911–11.847	0.001
PLAGL2	3.965	1.209–13.002	0.023	4.518	1.306–15.633	0.017

**Table 4:** Prognostic value of PLAGL2 expression for the OS revealed by univariate and multivariate analyses.

	Univariate analysis			Multivariate analysis		
Variables	Exp(B)	95%CI	p-value	Exp(B)	95%CI	p-value
Age	1.520	0.729–3.169	0.264			
Gleason	1.743	0.709–4.287	0.226			
T stage	2.200	1.071–4.520	0.032	2.167	1.013–4.636	0.046
PSM	1.481	0.692–3.171	0.312			
PSA	0.947	0.451–1.988	0.886			
Extraextension	2.835	1.344–5.980	0.006	2.582	1.192–5.597	0.016
SV invasion	11.537	4.163–31.970	0.000	10.044	3.531–28.568	0.000
PLAGL2	5.079	1.209–21.343	0.026	5.830	1.323–25.693	0.020

## Discussion

PLAGL2 has been shown to have both oncogenic and tumor suppressor activities in different tissues. So far as we know, no study has reported the precise role of PLAGL2 expression in PCa. We were interested to see whether there was any aberrant expression of PLAGL2 in PCa, and what role PLAGL2 played in PCa development and prognosis

In the present study, PLAGL2 DNA copy number or mRNA levels were firstly examined to analyze the difference between PCa and normal prostate tissues with available public microarray data. We found that PLAGL2 was significantly overexpressed in PCa patients compared to normal prostate, and the PLAGL2 expression was higher in metastatic PCa than in primary PCa. Our results were consistent with the previous reports that PLAGL2 genome copy number changed in some tumors. The chromosome location of PLAGL2 was a high recurrence-focal locus for gene amplification in the colon cancer [18]. Regional gains encompassing PLAGL2 gene were also confirmed in glioblastoma, AML samples and the immortalized lung epithelial cell lines [11, 16, 26]. Given these results, we speculated that PLAGL2 might be unregulated in PCa and play a potential role in the PCa disease.

To validate the hypothesis, qRT-PCR and western blot were employed to examine the expression levels of both the PLAGL2 mRNA and protein in paired PCa and adjacent normal prostate tissues as well as four prostate cell lines. The results supported that the PLAGL2 mRNA and protein expression were clearly elevated in PCa tissues. Analysis of PCa cell lines further showed that the mRNA and protein levels of PLAGL2 were upregulated in all three prostate cell lines, especially in more aggressive DU145 cells and PC-3 cells, compared to RWPE-1 cells. Additionally, in a cohort of PCa and BPH samples examined by IHC, high expression of PLAGL2 was frequently observed in PCa samples, while most of BPH samples showed low expression. Together, all the data strongly supported the notion that PLAGL2 was overexpressed in PCa.

There were studies which had already demonstrated that the upregulated PLAGL2 expression possessed the oncogenic capacity in tumors. PLAGL2 could transform NIH3T3 cells [27, 28], and might participate in AML development in cooperation with other fusion genes [11]. Recent reports further demonstrated that the elevated PLAGL2 expression was associated with the depth of colorectal tumor invasion [19], and female patients with lung adenocarcinoma who had low PLAGL2 expression and was at an early stage of disease had better prognosis [17]. Thus, further investigation was required to determine whether PLAGL2 overexpression could play a role in the development of PCa. We analyzed the relationship between PLAGL2 expression and clinicopathological characteristics of PCa patients. The increased PLAGL2 expression was only associated with higher preoperative PSA. However, the current study showed patients of high PLAGL2 expression, as well as high clinical stage, seminal vesicle invasion, and extraextension, had significantly poor five-year BCR-free survival and OS. Furthermore, multivariate analysis demonstrated that PLAGL2 expression was an independent prognostic factor for BCR-free survival and OS. Given these results, high PLAGL2 expression was correlated with poor prognosis independent of other factors.

To date, although there were some possible explanations about the acting mechanism of PLAGL2, the PLAGL2-induced oncogenesis was not completely understood. PLAGL2 inhibited neural stem cell differentiation and promoted self-renewal, partially through Wnt/β-catenin signaling [16]. PLAGL2-expressing NIH3T3 cells were tumorigenic in nude mice, and mitogenic and transforming potential of the cells was partly attributed to the activation of IGF-II pathway by PLAGL2 [29]. Conversely, a recent study demonstrated PLAGL2 could provoke cell apoptosis in response to hypoxia or a low serum medium [30]. The PLAGL2 could also inhibit the human myelomonocytic U937 cell proliferation via activation of p73 and p73 target genes, as a tumor suppressor gene [22]. The limited number of studies analyzing molecular mechanisms of PLAGL2 suggested that PLAGL2 was expressed distinctly in different cancers, and it probably had tissue specificity. And the contrary PLAGL2 function might owe to the different modulatory mechanism. Thus, the mechanism of the increased PLAGL2 expression contributing to PCa tumorigenesis required further investigation.

There were some limitations in our study. A large sample size and randomized study of PCa patients was needed to investigate the PLAGL2 expression and its correlation with PCa tumor development and prognosis. The function of PLAGL2-induced oncogenesis in PCa was still unknown and further study was needed to elucidate the molecular mechanism of the action.

To our knowledge, this was the first time that the common up-regulated expression of PLAGL2 was identified in human PCa compared to normal prostate. Furthermore, increased expression of PLAGL2 was strongly correlated with some adverse clinicopathological characteristics in PCa. Our study also provided the clinical evidence that PLAGL2 was independently prognostic for BCR-free survival and OS in PCa following radical prostatectomy. In conclusion, PLAGL2 may serve as a potential marker in the diagnosis and prognostic of PCa.
